# Poisoning by Fowler’s Solution

**Published:** 1887-08

**Authors:** 


**Poisoning by Fowler's Solution.**—Dr. J. P. Walker, in *Peoria Medical Monthly*, writes: "Mrs. D——'s child, aged about thirty months, climbed upon a chair and got an ounce vial from the cupboard containing Fowler's Solution and syrp. simp. aa ½ fl. ounce, of which about two drachms had been used by the mother for chronic ague, of which the child drank all about two drachms.

May 19, 1887—Saw the child within thirty minutes after drinking it. Condition of patient, pulse 60, respiration 12, skin cold, bluish; perspiration in great drops over the body; pupils dilated to their full extent.

Hot water, eggs and ipecac were given, and vomiting soon occurred. Hydrate sesquioxide ferrum was given as soon as could be made, in 30 grain doses, every twenty minutes.

May 21.—Child rapidly convalescing.

May 24.—All right now.

Think the success of the case depended greatly upon the prompt assistance and help of my students, Messrs. McLemore and Payne.

